# Special Section on Selected Topics in Biophotonics: Translating Novel Photonics Technology into Clinical Applications

**DOI:** 10.1117/1.JBO.28.12.121201

**Published:** 2023-11-29

**Authors:** Stefan Andersson-Engels, Peter E. Andersen

**Affiliations:** a Tyndall National Institute, Cork, Ireland; b University College Cork, Department of Physics, Cork, Ireland; c Technical University of Denmark, Department of Health Technology, Kongens Lyngby, Denmark

## Abstract

The editorial introduces the JBO Special Section on Selected Topics in Biophotonics: Translating Novel Photonics Technology into Clinical Applications.

## 1 Introduction

This JBO special section entitled “Translating Novel Photonics Technology into Clinical Applications” comprises one invited perspective paper, one invited tutorial review paper, and five contributed papers within the general scope of the Biophotonics ‘23 summer school (www.biop.dk). This section is meant to reflect highlights from the summer school and serves as an opportunity for participants to publish the work discussed during the school in a collected fashion for everyone to read. After completing the Biophotonics ‘23 summer school with this special section, we are very much looking forward to Biophotonics ’25.

## 2 Motivation and Purpose of Biophotonics Graduate Schools

The purpose of the “International Graduate Summer School: Biophotonics” is to provide education for postgraduate students at the highest international level. The school has so far always experienced higher interest than the number of students we can accommodate, illustrating the needs it fills in the field, and we have therefore implemented a peer-reviewed selection procedure. Apart from learning from renowned lecturers and scientists, the international atmosphere in having about 80 biophotonics scientists from across the world for a week in a confined space, on the beautiful small island Ven in Sweden, makes networking opportunities phenomenally good. The school certainly has the potential to create lifelong friendships, and to help advance the field by exchange of ideas. Lecturers are invited to teach at the school based on their scientific merits and pedagogical skills, and they reflect an international and diverse scientific community of highest quality. They are all encouraged to stay for a minimum of four days during the week-long school to facilitate informal discussions with participants around their lectures, expertise, and experiences. Students are requested to present their research in poster sessions early in the school week, providing ample opportunities to get to know all participants and their research. Lectures are scheduled daily before lunch and after dinner, providing afternoons for sight-seeing around the island, visiting the museum of the famous astronomer Tycho Brahe (1546-1601), sports activities, or smaller workshop on specified topics (e.g., general topics such as equity, diversity, and inclusion, or career opportunities; technical topics such as Monte Carlo simulations; or clinical perspective topics). This format sets a busy schedule during the week, while also allowing for informal discussions during the school.

## 3 Papers in the Special Section

This JBO special section comprises papers covering a wide variety of biophotonics techniques and applications. Brian Pogue provides a very interesting perspective on optical imaging in medicine and compare this field with the field of radiological imaging (https://doi.org/10.1117/1.JBO.28.12.121208). From the data he is presenting, both the market size and the number of medical doctors using imaging in their practice is considerably larger for optical than for radiological imaging. Even if it can be interesting to discuss the detailed inclusion criteria for both techniques studied, the difference is so manifest that the main conclusion cannot be overlooked. This is obviously fascinating, especially as most people probably would understand medical imaging to basically be synonymous with radiological imaging. In the paper Pogue also compares the funding situation for both these fields, and here the situation is the opposite, radiological imaging attracts significantly more research funding than optical imaging. He initiates thereby with this paper a discussion about how our field in biomedical optics can be more unified and harmonized to increase its visibility and success in attracting research funding.

A comprehensive tutorial review of the photoacoustic field is presented by Riksen et al. (https://doi.org/10.1117/1.JBO.28.12.121205). In this paper they discuss the physics of the imaging, the instrumentation requirements, and standardization, and give some practical examples that can be useful especially for researchers entering into this field. Moreover, we believe this paper would be very useful inspiration for anyone considering translating photoacoustic into any clinical application.

For interstitial photon time-of-flight measurements, it could potentially be very helpful to use the same fiber as both light sender and receiver, as only one fiber is needed and the source–detector distance would be well controlled. The large dynamic range in such signal may, however, be difficult to manage. Damagatla et al. investigate this problem from a medical perspective, a very compelling concept in detail their intriguing paper (https://doi.org/10.1117/1.JBO.28.12.121202).

Another paper, by Svea Steuer et al., investigates the human tympanic membrane with polarization-sensitive optical coherence tomography (OCT) imaging (https://doi.org/10.1117/1.JBO.28.12.121203; featured on the issue cover). They studied in this way the collagen fiber layer within the membrane. While they show it is feasible to image the collagen with PS-OCT, they conclude that further studies are required to validate whether this can be used to distinguish healthy and pathologically altered tympanic membranes.

Another interesting paper included in this section describes low-frequency oscillations in speckle contrast diffuse correlation tomography signals from measurements in piglets. Mohtasebi et al. conclude here that when it comes to brain injuries the knowledge of any spontaneous low frequency oscillation in the cerebral blood perfusion adds value in understanding the underlaying pathological mechanisms (https://doi.org/10.1117/1.JBO.28.12.121204). Measurements of low frequency oscillations of the cerebral blood perfusion could thereby be critical for neurological studies in for instance neonatal intensive care units.

Rocha et al. describe the development of an endoscope for early detection of malignancies in the fallopian tubes of the ovary (https://doi.org/10.1117/1.JBO.28.12.121206). Early detection of ovarian cancer has shown to double the 5-year survival rate. Both multispectral autofluorescence imaging and optical coherence tomography are included in this novel instrument. In particular, the authors in this paper describe the iterative prototyping employed to adopt the device to fit into the medical workflow and to optimize its functionality and robustness. The knowledge and acquired experience shared in this paper may inspire or guide other researchers considering similar translational research.

A relatively new field in biophotonics is guidance of orthopaedic procedures. Emerging optical sensing promises fewer side effects with new, more effective approaches aimed at improving patient outcomes following orthopaedic surgery. Li et al. here describe a comprehensive framework for selecting wavelengths with high differentiating potential for various tissue types in orthopaedic surgery (https://doi.org/10.1117/1.JBO.28.12.121207). The authors provide both the collected raw data and the code for the framework free to use for all.

